# Pericementitis

**Published:** 1882-07

**Authors:** 


### PERICEMENTITIS.

Dr. G. A. Mills read a paper upon this subject before the Medical Society of the County of Kings, on May 18th, of which we make the following summary:

No correct knowledge of the disease in question seems to have been current in the profession until the studies of Dr. John M. Riggs, of Hartford, were made public. The originality of these studies lead to the designation of "Riggs' disease" for the affection so well described, and for which Dr. Riggs had devised a method of treatment which in the hands of a skilled operator yields very satisfactory results.

The literature of the subject was added to by Drs. Mills, Reinwinkle, Niles and Bödecker, the latter having made original studies into the histology of the disease. Pericementitis is an expression of a greater or less degree of debility resultant upon nerve degeneracy. It has a variety of phases, yet is more generally manifested at the peripheral margin of the gum. This is characterized sometimes by a slight tinge of congestion, changing the appearance from a normal, pinkish color to one of deeper red or purple, and at others to an anæmic or bloodless, or colorless appearance. This is followed by a detaching, or relaxing the constriction of the membrane about the neck of the tooth. This is followed by the appearance of foreign substances, or by the absence of them, and an extended detachment of the tissues about the whole or a part of the neck of the tooth. The character of the inflammation—be it acute or otherwise, destructive or less so—is determined by the constitutional powers of resistance or the opposite. Some attribute the cause to the presence of deposits of lime and their admixtures (so-called tartar). Some claim that these are the results of inflammatory action, and still others call them sanguinary deposits, or residuum of the broken down tissues, blood, etc. While I have referred to the more general manifestations of this disease I have not noticed that there are many exciting causes, viz.: mechanical irritations, dead pulps, alveolar abscess, crowded conditions of the teeth, accumulations of foreign substances, uncleanliness, etc., etc. Not a few cases manifest a peculiar phase, noticed particularly associated with the exhibit of a recession of gum-tissue and not any inflammatory action apparent. These are denominated atrophy of the gum. It is thought by many to be caused by friction of the brush. While this might, under some circumstances, facilitate the loss of tissue, yet it is far from being the cause. This phase is seen at points where the brush would fail to have any such effect, viz.: not only on the labial and buccal, but on the lingual and palatal surfaces of the teeth ; and I would add that in many of these cases there is no perceptible presence of deposits of lime.

You will notice that I have. in passing, pointed out the manifestations of the disease in their mildest expressions. Starting out with the familiar adage that prevention is better than cure, it becomes of decided importance to emphasize familiarity with incipient stages, for if intelligence is active at this stage, we have under control the staying of its future destructiveness. The *serious* effects of this disease upon the general health are so well known to those who have familiarized themselves by an earnest and vigorous study of its workings, that it would be a crime to sit in silence and not proclaim the agonies associated. I am satisfied that large numbers are being cut off from their pilgrimage here premaurely, while thousands are dragging out a drooping existence of lassitude, depression and inanition directly and indirectly traceable to this disease. Perhaps I cannot do better than to state a case which will serve the purpose of demonstrating the many.

In the fall of 1878, Dr. Mason, Sr., Pres. of the Long Island Medical College, called at my office and consulted me about a patient of his who was in a wretched and rapid state of decline of health. He said he and his son had exhausted their remedies upon this patient, and he, having seen my articles published in Brochure, had become impressed that possibly this patient was a victim to the disease I had called his attention to. Several dentists had been consulted, but not with encouragement, excepting the extraction of the teeth. The patient came into my hands. She was about thirty-eight years of age, strong, nervous-bilious temperament, married. I found her with a dry, parched skin, feeble pulse, loss of appetite, depressed—sadly—sleepless, nausea on waking in the morning, and great loss of nerve energy. She had twenty-nine beautifully formed teeth, so loose that she had not been able to masticate with any power for a long time—some two years I think. From every socket was exuding a fetid discharge and very copious, so much so that she was obliged to place two large napkins under the side of her face to receive this flow at night while she slept. Now, this case did not prove to be one of suppurative pericementitis alone; it had involved the osseous formations, and that portion involving the sockets of the teeth was more or less destroyed. This case proved in treatment the necessity of surgical attention in the direction claimed by Dr. Riggs. It also proved that it had not been developed altogether during the time she had become cognizant of it, but circumstances of such severity had fastened upon her and so checked the activities of her organization that it was left with an enfeebled power to cope with the disorder already present. The patient being so highly organized, her sufferings were of the acute order, and played great havoc among the distributions of the sensory nerves. The result of the treatment in this case, both surgical and constitutional, brought the patient again into the sphere of activity and usefulness. To use her own words, "she was as good as new." The exciting cause of the rapid decline of this patient was a *terrific mental grief*. I could detail numerous cases that have come under my notice during the last eight years, particularly where a variety of associate disorders have become complicated. I do not need to pursue the enumeration of these facts. You are familiar with many instances where the progress of disorders frequently reveal before unknown ones, resulting in prolonged distress, and not uncommonly loss of life.

And now I do not think I need to go further, for I do not doubt that the intelligent mind will grasp the proper measure of the truths to which I have called your attention, and which are readily demonstrated in the oral cavities of ninety per cent of the people, in a greater or less degree of activity. I will not leave you with the impression that the specialty I represent is wholly alive, or in a large sense cognizant of the nature or of the destruction that is travelling madly over their every-day practice. As yet dentistry, as practised by the masses, can claim to be no more than Webster gives them credit for: "one who repairs teeth."

Gentlemen, it is from your ranks that much aid can come to assist those that are zealously devoting their energies to raise this special feature—oral surgery—to its sphere of greater usefulness in the alleviation of human suffering. And if you have been convinced, by what I have brought to your attention, of a conception of its importance, then I will not have spoken in vain.

